# Frequency of Going Outdoors as a Good Predictors for Incident Disability of Physical Function as well as Disability Recovery in Community-Dwelling Older Adults in Rural Japan

**DOI:** 10.2188/jea.16.261

**Published:** 2006-11-03

**Authors:** Koji Fujita, Yoshinori Fujiwara, Paulo H. M. Chaves, Yutaka Motohashi, Shoji Shinkai

**Affiliations:** 1 Research Team for Social Participation and Health Promotion, Tokyo Metropolitan Institute of Gerontology.; 2 Center on Aging and Health, Departments of Medicine and Epidemiology, Johns Hopkins University.; 3 Department of Public Health, Akita University School of Medicine.

**Keywords:** frequency of going outdoors, functional transition, predictor, community-dwelling elderly

## Abstract

**BACKGROUND:**

The clinico-epidemiologic relevance of the reduction in the frequency of going outdoors in older adults has not been well characterized. This study examined whether the frequency of going outdoors has predictive values for incident physical disability and recovery among community-dwelling elderly.

**METHODS:**

One thousand, two hundred and sixty-seven persons aged 65+ years who lived in a rural community in Niigata, Japan, and participated in the baseline survey were assessed again 2 years later in terms of mobility, and instrumental and basic activities of daily living (IADL and BADL). We compared the incident disability and recovery at follow-up among three subgroups classified by the baseline frequency of going outdoors: once a day or more often, once per 2-3 days, and once a week or less often. Multivariate analyses tested associations between the frequency of going outdoors and functional transition, independent of potential confounders.

**RESULTS:**

A lower frequency of going outdoors at baseline was associated with a greater incident disability, and a lower recovery at the two-year follow-up. Even after adjustment, the effects of going outdoors remained significant. Adjusted risks of incident mobility and IADL disabilities were significantly higher (odds ratio[OR]=4.02, 95% confidence interval [CI]: 1.77-9.14 and OR=2.65, 95% CI: 1.06-6.58), respectively, and recovery from mobility disability was significantly lower (OR=0.29, 95% CI: 0.08-0.99) for “once a week or less often” subgroup compared with “once a day or more often” subgroup.

**CONCLUSION:**

The frequency of going outdoors is a good predictor for incident physical disability and recovery among community-living elderly. Public health nurses and clinicians should pay more attention how often their senior clients usually go outdoors.

Older people go outdoors for various purposes, such as going shopping, taking a walk, visiting friends, and working in their garden or field. These activities are important for the maintenance of health and quality of life. With advancing age, there is a decline in the frequency of going outdoors. For example, in Japan more than 50% of older people aged 65-69 years go outside the house at least once a day, but among those aged 70-79 years and over 80 years, the respective percentages are less than 40% and 30%.^1^ The clinico-epidemiologic relevance of the reduction in the frequency of going outdoors in community-dwelling older adults has not been well characterized.

Getting outdoors requires a certain level of physical and mental functions. Not surprisingly, the decline in the frequency of going outdoors with aging may be linked to concomitant deterioration in health status.^2^^-^^4^ The extent to which reduction of the frequency of going outdoors merely reflects the presence of physical disability, whether mismatches may occur (e.g., reduced frequency of going outdoors without advanced decline in physical function, or no significant reduction in the frequency of going outdoors despite the presence of advanced physical disability), and the prognostic value of such mismatches remain to be determined.

Additionally, because the frequency of going outdoors is directly linked to levels of physical activity and social engagement among community-dwelling older people,^4^^,^^5^ which are important for physical and mental functioning in older persons,^6^^-^^10^ it can be hypothesized that an increased frequency of going outdoors could actually help preserving functioning in later life. To our knowledge, only one recent longitudinal study^11^ examined the association between the frequency of going outdoors and functioning in later life. This study reported that ambulatory frail elders going outdoors more often were more likely to maintain physical and mental functioning than those going outdoors less often. Consistently, Simonsick et al^12^ demonstrated that getting outside and walking are important in maintaining the mobility of frail older women, though the frequency of going outdoors was not specifically examined in the study. Taken together, these studies suggest that going outdoors might help to preserve functioning in a certain subset of older people, i.e., functionally-disabled, frail elders. However, studies conducted in disabled populations are particularly prone to biased results favoring the association between reduced frequency of going outdoors with subsequent disability, as a consequence of selection bias; i.e., there may be an over-representation of frail individuals at high risk of progression of disability among those with reduced frequency of going outdoors. In this context, examining the role of getting outdoors in more competent elders may provide further insight.

Additionally, previous studies have not looked at another aspect of functional transition, i.e., recovery from disability, and going outdoors. It has been documented that even if older people become disabled, a substantial proportion of them recovers independence.^13^^,^^14^ Examining the role of getting outdoors in functional recovery may offer further insight into disablement process, and may prove useful for the development of novel screening and targeted intervention preventive opportunities.

The overall objective of the current study was to describe the distribution of the frequency of going outdoors in a population-based sample of community-dwelling older adults living in rural Japan, and document potential heterogeneity in terms of clinical and physical function variables across categories reflecting frequency of going outdoors. Furthermore, we tested the hypothesis that a reduced frequency of going outdoors would independently predict incident disability, as well as recovery from disability, in a dose-response fashion, in three domains of physical function: mobility, instrumental activities of daily living (IADL), and basic self-care domain.

## METHODS

### Study Population

This was a two-year prospective study conducted in Yoita Town, a rural area in Niigata Prefecture, Japan. In 2000, the total population of this town was 7,493, and the proportion of older adults (i.e., 65 years and older) was 22.3%. A baseline survey was conducted in November 2000, in which all residents aged 65+ years (n=1,673) were invited to participate. Out of them, 1,544 (92.3%) persons responded to the survey. Among the 129 non-responders, 80 were institutionalized, 2 were relocated, 3 had died, and 44 refused to participate.

A follow-up survey was conducted in October of 2002. Out of the initial 1,544 respondents, 1,283 (83.1%) participated again. Among the 261 non-participants, 57 were institutionalized, 12 persons were relocated, 81 persons had died, 88 refused to participate, and the remaining 23 persons did not participate for miscellaneous reasons.

Trained personnel conducted standardized, face-to-face interviews with study subjects either at the community hall or in their homes, both at baseline and follow-up. The purpose of the study (collecting information on how to prevent disability among older people) was explained before interview, and all subjects participated voluntarily in accordance with a protocol approved by the Ethics Committee of Tokyo Metropolitan Institute of Gerontology.

### Variables

The questionnaire comprised of sociodemographic, medical and physical function, cognitive, and psychosocial items. Sociodemographic variables included age, sex, education, principal occupation, and household structure. Medical and physical function variables included the basic activities of daily living (BADL), IADL, walking ability, visual and hearing ability, pain, chronic medical conditions, urinary incontinence, use of outpatient clinics, history of hospital admission, and frequency of going outdoors. Psychosocial variables included cognitive function, subjective health, contacts with intimate friends or relatives, participation in formal or informal community groups, and depressive mood state.

In regard to the assessment of the frequency of going outdoors, subjects were asked the question, “How often do you usually go outside the house?” (Examples of going outdoors include going shopping, taking a walk, going to a hospital, or going out to work in your garden or field). The frequency was categorized as: (1) once a day or more, (2) once per 2-3 days, or (3) once a week or less often.^4^

### Outcome Measures

Outcome measures included incident disability and recovery from disability at 2-year follow-up in mobility, IADL, and BADL. Mobility was assessed by the questions: “Can you walk 1 km on a level surface without difficulty?” and “can you climb up stairs without difficulty?”.^15^^,^^16^ We scored 2 for the answer “able to do so without difficulty”, 1 for the answer “able to do so, but with difficulty”, and 0 for the answer “unable to do so.” Summed scores for the two items gave a mobility score of 0-4, a high score indicating good mobility. This assessment has been validated against maximal walking speed^17^ in 918 community-living older subjects (Pearson’s correlation coefficient between the two measures = 0.567, p<0.001]. For our present purpose, mobility disability was defined as a subject who answered “unable to do so” to at least one of the two questions.

The IADL was measured using the five-item subscale of Instrumental Self-Maintenance of the TMIG Index of Competence:^18^ using transportation, going shopping, preparing meals, paying bills, and handling one’s own banking. We defined IADL disability as a subject reporting that he/she could not undertake one or more IADLs.^19^ We asked subjects about their dependence with respect to five BADLs: eating, dressing, toiletry, bathing, and walking on a level surface.^20^^,^^21^ We defined BADL disability as a subjects reporting that he/she was dependent in one or more BADLs.

### Confounding Factors

Demographic, physical, medical, and psychological factors previously associated with the frequency of going outdoors, independent of mobility or functional status were included in the current study, specifically age, sex, chronic medical conditions,^2^^,^^22^^,^^23^ impairments of vision and hearing,^3^^,^^22^ urinary incontinence,^3^^,^^24^^-^^26^ subjective health,^2^ depressive mood state^2^^,^^23^ and cognitive function.^4^

We ascertained the presence of chronic conditions (defined as a history of physician-diagnosed heart disease, stroke or diabetes mellitus) from the subject’s report. In addition, we defined arthritis as persistent pain in any joint of the arms, hips, or legs and included it among the chronic conditions. The total number of chronic conditions was calculated (range, 0-4) and was used as an arbitrary index of comorbidity. Urinary incontinence was defined as “present” when a subject reported that urine sometimes leaked following an urge to void. Impairment of vision or hearing was defined as “present” if a subject reported some difficulty in reading a newspaper or communicating verbally with others, respectively. Depressive mood was assessed by the 15-item short version of the Geriatric Depression Scale (GDS).^27^^,^^28^ Higher scores in the scoring range of 0-15 indicated a greater tendency to depression; the cut-off point was set at 5/6.^28^^,^^29^

As to subjective health, subjects rated their health as excellent, good, fair, or poor.^30^^-^^32^ Low subjective health was defined by a rating of fair or poor. Cognitive function was assessed using the Mini-Mental State Examination (MMSE).^33^^,^^34^ Scores ranged from 0 to 30, higher score indicating better function. Cognitive impairment was defined as an MMSE score <20, based on previous work.^35^

### Statistical Analyses

The baseline characteristics and trajectory over time were compared among subgroups classified by the frequency of going outdoors. P-value for trend was assessed by Kendall rank correlation coefficient for categorical variables as well as continuous variables. The incident disability or recovery from disability was compared among subgroups stratified by the frequency of going outdoors within initially nondisabled individuals or disabled individuals, respectively. We performed logistic regression analyses to explore independent associations between the frequency of going outdoors at baseline and functional transition, controlling for potential confounders listed above. We used SPSS^®^ 12.0J for Windows for all analysis, and accepted a two-tailed probability level <0.05 as statistically significant.

## RESULTS

### Baseline Characteristics and Follow-up Status

Of the 1,522 older persons who participated in the baseline survey and gave data on the frequency of going outdoors, 1,161 (76.3%), 200 (13.1%), and 161 persons (10.6%) reported their frequency of going outdoors as once a day or more, once per 2-3 days, and once a week or less, respectively.

Baseline characteristics differed significantly among these three groups (Table 1). The lower the frequency of going outdoors was, the greater the prevalence of functional limitation and impairment was. Depressive mood, poor subjective health, and low cognition were also more prevalent among those with a lower frequency of going outdoors. Furthermore, there was evidence of substantial mismatch between degree of disability and frequency of going outdoors. For example, among those with an intermediate reduction of the frequency of going outdoors (once per 2-3 days), more than 70% of the subjects were classified as having no mobility disability, and more than half of the study subjects had no IADL disability. Among those in the group with the most reduced frequency of going outdoors (once a week or less), less than 50% had BADL disability.

**Table 1.** Baseline characteristics of the subjects according to the frequency of going outdoors.

Characteristics	Frequency of going outdoors			p-value for trend*

	once a day or more often	once per 2-3 days	once a week or less often	
	(n=1,161, 76.3%)	(n=200, 13.1%)	(n=161, 10.6%)	
Sex, Female, n(%)	694 (59.8)	122 (61.0)	99 (61.5)	0.618
Age, mean(standard deviation)	73.5 ( 6.0)	76.8 ( 7.8)	80.4 ( 7.7)	<0.001

Prevalent chronic medical condition				
Stroke, n(%)	73 ( 6.3)	37 (18.5)	36 (22.4)	<0.001
Heart Disease, n(%)	172 (14.8)	43 (21.5)	42 (26.1)	<0.001
Diabetes Mellitus, n(%)	172 (14.8)	27 (13.5)	19 (11.8)	0.279
Arthritis, n(%)	468 (40.3)	81 (40.5)	63 (39.1)	0.860
Prevalent other chronic condition				
Visual impairment, n(%)	117 (10.1)	51 (25.5)	42 (26.3)	<0.001
Hearing impairment, n(%)	175 (15.1)	52 (26.0)	58 (36.3)	<0.001
Urinary incontinence, n(%)	94 ( 8.1)	41 (20.5)	63 (39.1)	<0.001
Depressive mood, GDS-sv>5, n(%)	211 (19.0)	72 (40.9)	60 (47.2)	<0.001
Self-rated health, fair or poor, n(%)	311 (26.9)	79 (41.1)	77 (54.2)	<0.001
Use of outpatient clinic in the previous month, yes, n(%)	933 (80.4)	154 (77.0)	130 (81.3)	0.639
History of hospitalization in the previous year, yes, n(%)	91 ( 7.8)	25 (12.5)	30 (18.6)	<0.001

Physical Function				
Walking ability over 1km, n(%)				<0.001
able, no difficulty	829 (71.5)	99 (49.5)	23 (14.4)	
difficult	283 (24.4)	50 (25.0)	46 (28.8)	
unable	48 ( 4.1)	51 (25.5)	91 (56.9)	
Climbing up stairs, n(%)				<0.001
able, no difficulty	849 (73.2)	100 (50.0)	34 (21.3)	
difficult	285 (24.6)	68 (34.0)	51 (31.9)	
unable	26 ( 2.2)	32 (16.0)	75 (46.9)	
Mobility disability, n(%)	58 ( 5.0)	56 (28.0)	99 (61.5)	<0.001

IADL disability, n(%)	183 (15.8)	83 (41.5)	125 (78.1)	<0.001
BADL disability, n(%)	31 ( 2.7)	43 (21.8)	79 (49.4)	<0.001

Cognitive Function				
MMSE score, median	26.8	25.8	23.4	<0.001
MMSE<20, n(%)	42 ( 3.6)	26 (13.3)	39 (25.5)	<0.001

IADL: instrumental activities of daily living; BADL: basic activities of daily living; MMSE: Mini-Mental State Examination; GDS-sv: short-version of geriatric depression scale

*: P-value for Kendall rank correlation coefficient

Table 2 shows trajectories over time according to the frequency of going outdoors at baseline. Those who were initially going outdoors once a week or less were more likely to be institutionalized or deceased at follow-up when compared with the “once a day or more” subgroup.

**Table 2.** Trajectories over time of the subjects according to the frequency of going outdoors

Frequency of going outdoors at baseline	Status at 2-year follow-up			

	Community-dwelling	Hospital/nursing home	Deceased	Loss to follow-up*
Once a day or more often (n=1,161)	1,001 (86.2%)	32 ( 2.8%)	27 ( 2.3%)	101 ( 8.7%)
Once per 2-3 days (n=200)	166 (83.0%)	10 ( 5.0%)	12 ( 6.0%)	12 ( 6.0%)
Once a week or less often (n=161)	100 (62.1%)	12 ( 7.5%)	34 (21.1%)	15 ( 9.3%)

*: Loss to follow-up because of refusal, absence, relocation or other reasons.

### Incident Disability and Disability Recovery during Follow-up

The subjects who were alive and living at home over the two-year period were physically more competent at baseline compared with institutionalized or deceased persons at follow-up, or loss to follow-up persons (Table 3). Table 4 summarizes the functional transition over time among them according to physical function status and frequency of going outdoors at baseline. The lower the frequency of going outdoors at baseline, the greater the risk of incident disability in all three domains of physical function studied at follow-up among initially nondisabled individuals. Consistently, the lower the frequency of going outdoors at baseline, the lower the probability of recovery from disability at follow-up among initially disabled individuals. Also worth noticing were the substantial transitions into and out of disability presented by the group with intermediate reduction in the frequency of going outdoors (once per 2-3 days). For example, in this group, among those who did not have IADL disability at baseline, almost 17% developed incident IADL disability, which was 1.8 times higher than the group of subjects going outdoors at least once a day. Substantial disability recovery was also observed among those in the group with intermediate reduction in the frequency of going outdoors (once per 2-3 days). For example, in this group, among those with mobility disability at baseline, 26.3% recovered from disability. This probability was 1.8 times higher than that observed in the group with mobility disability who was going outdoors only once a week or less.

**Table 3.** Comparison of baseline characteristics between community-dwelling subjects, and deceased, institutionalized, or loss to follow-up subjects at 2-year follow-up.

Characteristics	Status at 2-year Follow-up		p-value*

	Community-dwelling	Deceased, insitutional- ized or loss to follow-up	
	(n=1,267)	(n=255)	
Sex, Female, n(%)	789 (62.3)	126 (49.4)	<0.001
Age, mean (standard deviation)	74.3 (6.6)	76.6 ( 7.6)	<0.001

Prevalent chronic medical condition			
Stroke, n(%)	105 ( 8.3)	41 (16.1)	<0.001
Heart Disease, n(%)	217 (17.1)	40 (15.7)	.323
Diabetes Mellitus, n(%)	182 (14.4)	36 (14.1)	.502
Arthritis, n(%)	523 (41.3)	89 (34.9)	.033
Prevalent other chronic condition			
Visual impairment, n(%)	159 (12.5)	51 (20.1)	.002
Hearing impairment, n(%)	216 (17.0)	69 (27.2)	<0.001
Urinary incontinence, n(%)	133 (10.5)	65 (25.5)	<0.001
Depressive mood, GDS-sv>5, n(%)	271 (22.6)	72 (33.6)	<0.001
Self-rated health, fair or poor, n(%)	380 (30.3)	87 (37.0)	0.025
Use of outpatient clinic in the past month, yes, n(%)	1021 (80.6)	196 (76.9)	0.099
History of hospitalization in the past year, yes, n(%)	113 ( 8.9)	33 (12.9)	0.034

Physical Function			
Walking ability over 1km, n(%)			<0.001
able, no difficulty	818 (64.6)	133 (52.4)	
difficult	326 (25.8)	53 (20.9)	
unable	122 ( 9.6)	68 (26.8)	
Climbing up stairs, n(%)			<0.001
able, no difficulty	852 (67.3)	131 (51.6)	
difficult	340 (26.9)	64 (25.2)	
unable	74 ( 5.8)	59 (23.2)	

Mobility disability, n(%)	138 (10.9)	75 (29.4)	<0.001
IADL disability, n(%)	277 (21.9)	114 (45.1)	<0.001
BADL disability, n(%)	87 ( 6.9)	66 (26.1)	<0.001

Cognitive Function			
MMSE score, median	26.6	24.7	<0.001
MMSE<20, n(%)	61 ( 4.8)	46 (18.6)	<0.001

IADL: instrumental activities of daily living, BADL: basic activities of daily living, MMSE: Mini-Mental State Examination, GDS-sv: short-version of geriatric depression scale

* : *χ*^2^ test for categorical data and Mann-Whitney U test for continuous data.

**Table 4.** Functional transition over time according to respective physical function status and frequency of going outdoors at baseline.

Physical function at baseline n (%)		Frequency of going outdoors at baseline	n (%)	Disability status at follow-up		P-value for trend*

				No disability	Disability	
Mobility	No Disability	Once a day or more	955 (100%)	900 (94.2%)	55 ( 5.8%)	
1267 (100%)	1,129 (89.1%)	Once per 2-3 days	128 (100%)	109 (85.2%)	19 (14.8%)	<0.001
		Once a week or less	46 (100%)	30 (65%)	16 (35%)	

	Disability	Once a day or more	46 (100%)	21 (46%)	25 (54%)	
	138 (10.9%)	Once per 2-3 days	38 (100%)	10 (26%)	28 (74%)	<0.001
		Once a week or less	54 (100%)	8 (15%)	46 (85%)	

IADL	No Disability	Once a day or more	853 (100%)	774 (90.7%)	79 ( 9.3%)	
1259 (100%)	985 (78.2%)	Once per 2-3 days	101 (100%)	84 (83.2%)	17 (16.8%)	0.002
		Once a week or less	31 (100%)	21 (68%)	10 (32%)	

	Disability	Once a day or more	143 (100%)	48 (33.6%)	95 (66.4%)	
	274 (21.8%)	Once per 2-3 days	62 (100%)	11 (18%)	51 (82%)	<0.001
		Once a week or less	69 (100%)	3 ( 4%)	66 (96%)	

BADL	No Disability	Once a day or more	965 (100%)	927 (96.1%)	38 ( 3.9%)	
1244 (100%)	1,160 (93.2%)	Once per 2-3 days	134 (100%)	120 (89.6%)	14 (10.4%)	<0.001
		Once a week or less	61 (100%)	49 (80%)	12 (20%)	

	Disability	Once a day or more	23 (100%)	14 (61%)	9 (39%)	
	84 ( 6.8%)	Once per 2-3 days	25 (100%)	6 (24%)	19 (76%)	0.005
		Once a week or less	36 (100%)	8 (22%)	28 (78%)	

IADL: instrumental activities of daily living, BADL: basic activities of daily living

*: p-value for Kendall rank correlation coefficient

### Independent Associations of Frequency of Going Outdoors with Incident Disability and Disability Recovery

Risks of incident disability in physical function among initially nondisabled individuals were calculated for “once per 2-3 days” and “once a week or less” subgroups relative to “once a day or more” subgroups (Table 5). The lower the frequency of going outdoors at baseline was, the greater the odds ratio (OR) for incident disability was. Adjustment for potential confounders reduced these associations, but the odds ratios of mobility and IADL disability for the “once a week or less” subgroup remained statistically significant (OR=4.02, 95% confidence interval [CI]: 1.77-9.14, and OR=2.65, 95% CI: 1.06-6.58, respectively). The effects of a reduced frequency of going outdoors were evident in regard to mobility, IADL and BADL disability.

**Table 5.** Logistic regression estimating the risk of incident disability in different domains of physical function among initially nondisabled subjects.

Frequency of going outdoors at baseline	Incident outcome at follow-up					

	Mobility disability		IADL disability		BADL disability	
		
	Crude OR	Adjusted OR*	Crude OR	Adjusted OR*	Crude OR	Adjusted OR*
	(95%CI)	(95%CI)	(95%CI)	(95%CI)	(95%CI)	(95%CI)
Once a day or more	1	(reference)	1	(reference)	1	(reference)

Once per 2-3 days	2.90	1.78	1.98	1.48	2.97	1.76
	(1.66-5.08)	(0.91-3.47)	(1.12-3.51)	(0.75-2.93)	(1.56-5.66)	(0.81-3.85)

Once a week or less	8.46	4.02	4.67	2.65	4.45	1.41
	(4.28-16.7)	(1.77-9.14)	(2.12-10.3)	(1.06-6.58)	(1.96-10.1)	(0.54-3.66)

Hosmer & Lemeshow *χ*^2^		6.38 (d.f.=8)		7.41 (d.f.=8)		11.20 (d.f.=8)
		P=0.604		P=0.493		P=0.190

IADL: instrumental activities of daily living, BADL: basic activities of daily living, OR: odds ratio, CI: confidence interval, d.f.: degree of freedom

* : Adjusted odds ratio for age, sex, mobility score, comorbidity, visual and hearing impairments, urinary incontinence, self-rated health, depressive mood, and cognitive function at baseline.

Likewise, the relative probabilities of recovery in physical function among initially disabled individuals were calculated for “once per 2-3 days” and “once a week or less” subgroups relative to “once a day or more” subgroups (Table 6). The lower the frequency of going outdoors at baseline, the lower the odds ratio for recovery (=lower chance of recovery). Even after adjustment for potential confounders, the odds ratio of recovery from mobility disability for the “once a week or less” subgroup remained statistically significant (OR=0.29, 95% CI: 0.08-0.99).

**Table 6.** Logistic regression estimating the likelihood of recovery from disability in different domains of physical function among initially disabled subjects.

Frequency of going outdoors at baseline	Incident outcome at follow-up					

	Recovery in mobility		Recovery in IADL		Recovery in BADL	
		
	Crude OR	Adjusted OR*	Crude OR	Adjusted OR*	Crude OR	Adjusted OR*
	(95%CI)	(95%CI)	(95%CI)	(95%CI)	(95%CI)	(95%CI)
Once a day or more	1	reference	1	reference	1	reference

Once per 2-3 days	0.43	0.59	0.43	0.90	0.20	0.37
	(0.17-1.07)	(0.18-1.97)	(0.20-0.89)	(0.35-2.30)	(0.06-0.70)	(0.04-3.12)

Once a week or less	0.21	0.29	0.09	0.37	0.18	0.22
	(0.08-0.53)	(0.08-0.99)	(0.03-0.30)	(0.09-1.57)	(0.06-0.58)	(0.02-2.21)

Hosmer & Lemeshow *χ*^2^		7.84 (d.f.=8)		9.89 (d.f.=8)		6.34 (d.f.=8)
		P=0.449		P=0.273		P=0.609

IADL: instrumental activities of daily living, BADL: basic activities of daily living, OR: odds ratio, CI: confidence interval, d.f.: degree of freedom

* : Adjusted odds ratio for age, sex, mobility score, comorbidity, visual and hearing impairments, urinary incontinence, self-rated health, depressive mood, and cognitive function at baseline.

## DISCUSSION

In this observational study, we found evidence of substantial heterogeneity in terms of the frequency of going outdoors and physical function status. For example, among older persons with a reduced frequency of going outdoors (“once per 2-3 days” or “once a week or less”), 43% did not have mobility disability, and 58% did not have IADL disability. On the other hand, a substantial proportion of those with mobility disability or IADL disability (27% and 47%, respectively), as well as 20% of those with ADL disability, went out once a day or more often.

The present study also demonstrated that the frequency of going outdoors has an independent prognostic value not only for prediction of incident physical disability, but also for disability recovery in community-dwelling older people. The prognostic value was most evident for mobility disability, followed by IADL disability, but that for BADL disability did not remain significant when controlled for potential confounders. Both mobility and IADL disabilities are well-known predictors for BADL disability among community-dwelling elderly. If we could follow up the cohort for a longer period, the frequency of going outdoors at baseline would become predictive for BADL disability.

Previous studies that examined the relationship of going outdoors and physical functioning were confined to the frail elderly.^11^^,^^12^ By studying higher physically functioning subjects, the present study comparatively minimizes the impact of reverse causality -- though certainly does not exclude it -- on the findings reported here. Additionally, this study adds to the external validity of previous findings (rural population vs. urban,^12^ Japan vs. USA^12^).

These results are also consistent with the hypothesis that those without mobility disability but having reduced their walking frequency are at a stage of preclinical disability and at high risk for mobility disability.^36^^,^^37^ Additionally, reduced frequency of going outdoors may well represent reduced life space, which has been supposed to be a risk factor for subsequent physical disability.^38^ Examining an analogy of reduced frequency of going outdoors and preclinical disability, or reduced life space warrants for future studies.

The results presented here should be interpreted in light of important limitations. First, one has to consider the possibility of selection bias favoring an association between reduced frequency of going outdoors and increased risk of incident physical disability. This could have occurred in the case of an over-representation of subjects at a particularly high risk of incident physical disability in the group of subjects who self-selected to go outdoors less often. For example, it has been documented that among persons who do not have mobility difficulty, there exists a broad range of mobility capacity.^37^^,^^39^ It is thus possible that persons going outside daily had higher functional capacity and lower likelihood of preclinical disability at baseline than those who did not leave the home as frequently.

Second, the possibility of residual confounding by severity of disease, physiologic impairment, and functional limitation, explaining at least partly our results should be considered. More sensitive measures for grading disease severity and physical function might be needed, though it would lead to attenuate the association between the frequency of going outdoors and physical disability or recovery.

Third, the over-fitting issue should be considered, especially for disability recovery. For example, it is recommended that more than 10 events per independent variable are included in the logistic regression model;^40^ otherwise, risk indicators may be unreliable. However, judging from the goodness-of-fit statistics, the models were satisfactorily fit for estimating disability recovery.

Fourth, a certain degree of misclassification of the frequency with which study subjects reported they went outdoors should be expected, particularly if one considers that such a frequency may have varied over the year as a function of changes in the weather, and/or acute and chronic changes in health status. To address this issue, we examined the consistency of response every three month over two years among a sample of older subjects (n=243) using an outpatient clinic in the present study area. The frequency of going outdoors showed a slight seasonal-variation, but two adjacent data were satisfactorily consistent (Kendall *τ*=0.621 between June-August and September-November data). Among subjects classified as “once a day or more”, 12% (18/147) reported going outdoors “once per 2-3 days” three month later; of those classified as “once per week or less”, 20% (6/30) reported going outdoors “once per 2-3 days” or “once a day or more” three month later. Thus, it is possible that our results might have been affected by misclassification bias. In the case of a non-differential or even differential (e.g., if subjects with preclinical disability were more likely to reduce their frequency of going outside as a result of the weather and/or acute changes in health status than the more robust, high-functioning counterparts) misclassification, the results presented here would have been biased towards an underestimation of the true risk of disability associated with a reduced frequency of going outdoors.

Could the association between frequency of going outdoors and disability status be causal? We have recently reported that the frequency of going outdoors is significantly associated with physical activity level and the extent of social engagement among community-dwelling older Japanese.^4^^,^^5^ Appropriate levels of physical activity and social engagement have been documented to have advantages for maintaining physical function and mental health in later life.^6^^-^^10^ It is plausible to consider that increased frequency of going outdoors actually exert a protective effect on physical disability by way of increased physical activity and social engagement. Nonetheless, the possibility that frequency of going outdoors is merely a marker of other causal risk factors, such as disease burden and health status, cannot be excluded.

Our findings provide support for the hypothesis that going outdoors frequently might be an independent, protective factor against physical disability. Though it remains to be established whether the associations reported here are causal or non-causal, it should be noted that regular physical activity involving walking outdoors is an important health promotion strategy for primary prevention of physical disability in older populations. In this context, and taking into account that information on how often one goes outdoors can be easily obtained and has a remarkable predictive value, we hypothesize that such an assessment might provide a novel opportunity for clinical and public health disability-related screening and prevention. For example, such an assessment could potentially help increasing the awareness of health care professionals as to how often their senior clients go outdoors in daily life, as well as, could serve as an incentive for physicians and nurses to encourage their older patients to get outside the house more often for walking and other health promotion activities. The effectiveness of such a screening program should be evaluated by future research. Additionally, given its potential impact on the determination of the frequency with which older adults go outdoors, the investigation of the epidemiology of potentially relevant neighborhood factors is warranted.

In summary, the present study shows that the frequency of going outdoors is a good predictor for incident physical disability and recovery among community-living elderly. Public health nurses and clinicians should pay more attention how often their senior clients usually go outdoors.
